# Well-Known, Misidentified, or Unnamed? A DNA Barcode-Based Reassessment of the Lepidoptera Fauna of Cyprus Supported by Morphology

**DOI:** 10.3390/insects17010004

**Published:** 2025-12-19

**Authors:** Peter Huemer, Özge Özden, Erwin Rennwald, Ian Barton, Jari Junnilainen, Axel Hausmann, Erik J. van Nieukerken, Paul D. N. Hebert

**Affiliations:** 1 Naturwissenschaftliche Sammlungen, Sammlungs- und Forschungszentrum, Tiroler Landesmuseen Betriebsges.m.b.H., 6060 Hall in Tirol, Austria; 2 Faculty of Agriculture, Near East University, 99138 Nicosia, Cyprus; ozge.ozden@neu.edu.tr; 3 Independent Researcher, Mozartstr. 8, 76287 Rheinstetten, Germany; erwin@rennwald-biol.de; 4 Independent Researcher, 7 Cage Lane, Cambridgeshire CB6 3LB, UK; imbarton@btinternet.com; 5 Independent Researcher, Mahlapolku 3, 01730 Vantaa, Finland; junnilainen.jari@googlemail.com; 6 SNSB—Zoologische Staatssammlung München, Münchhausenstr. 21, 81247 München, Germany; hausmann.a@snsb.de; 7 Naturalis Biodiversity Center, P.O. Box 9517, 2300 RA Leiden, The Netherlands; erik.vannieukerken@naturalis.nl; 8 Centre for Biodiversity Genomics, University of Guelph, 150 Stone Road East, Guelph, ON N1G 2W1, Canada; phebert@uoguelph.ca

**Keywords:** checklist, Cyprus, DNA barcode, endemism, faunistics, Lepidoptera, taxonomy

## Abstract

This study presents the first comprehensive molecular analysis of the Lepidoptera fauna of Cyprus based on DNA barcoding. A total of 1859 DNA barcode sequences were generated, representing 701 Barcode Index Numbers (BINs) and thus putative species. Morphological examination enabled the assignment of 596 BINs to 580 Linnaean species. Based on this genetically validated species inventory—complemented by morphologically examined specimens and a critical review of the literature—a new checklist for the Lepidoptera of Cyprus is provided. In total, 1213 species are accepted as confirmed or considered likely based on published but unverified records. The checklist includes 57 genetically confirmed first records for Cyprus and 62 new records supported solely by morphology. Remarkably, 10 species are recorded as new to Europe: *Alloclita deprinsi*, *Cochylimorpha diana*, *C. additana*, *Pammene avetianae*, *P. nannodes*, *Cydia alienana*, *Ephestia abnormalella*, *Hypsotropa paucipunctella*, *Dysauxes parvigutta*, and *Bryophilopsis roederi*. In addition, 105 BINs could not be assigned to a species. Preliminary morphological assessment indicates that many of these represent cryptic taxa or belong to taxonomically unresolved species complexes. Furthermore, 35 morphology-based records could be identified at best to the genus level. The study also lists 158 previously published species that are now considered likely misidentifications and therefore excluded from the Cypriot fauna.

## Simple Summary

Lepidoptera (butterflies and moths) are among the most diverse yet most threatened groups of organisms in Europe. They play vital ecological roles as pollinators, decomposers, and an important food source for many species, and are often used as indicators in conservation assessments. In this study, we present the first comprehensive reassessment of the Lepidoptera fauna of Cyprus. Our analysis is supported by DNA barcode data covering approximately half of all recorded species. The discovery of more than 100 newly recorded species, over 100 currently unidentified taxa, and more than 10% misidentifications in the previous literature highlights major gaps in the existing faunal inventory. These findings underline the urgent need for comprehensive DNA barcode reference libraries and continued taxonomic revisions to ensure an accurate understanding of the island’s Lepidoptera diversity.

## 1. Introduction

Cyprus, with an area of 9251 km^2^, is the third-largest island in the Mediterranean, after Sardinia and Sicily. It is located in the eastern part of the Mediterranean basin, with a maximum east–west extent of 225 km and a north–south width of 90 km. Although the climate of Cyprus is influenced by its geographical position, it can generally be characterized as Mediterranean. Its main features are a short, mild, and rainy winter, and a long, hot summer [1].

The island is composed of four geological zones: (a) the Troodos Geotectonic Zone, (b) the Mamonia Geotectonic Zone, (c) the Kyrenia Geotectonic Zone, and (d) the Circum Troodos zone including the Mesaoria Plain. The 92-million-year-old ophiolitic rocks of the Troodos Zone, which represent a section of the oceanic crust and extend beneath the Mesaoria Plain, form the core of this geotectonic unit. These mountains reach heights of nearly 2000 m. The Mamonia Geotectonic Zone consists of allochthonous igneous rocks (serpentinites, pillow lavas), sedimentary rocks (sandstones, siltstones, mudstones), and, to a lesser extent, metamorphic rocks (recrystallised limestones, schists), ranging in age from 210 to 95 million years. The northernmost geomorphological unit of Cyprus is the Kyrenia Geotectonic Zone, characterised by a narrow, steep limestone mountain range reaching a maximum height of approximately 1000 m and extending from the Apostolos Andreas area in the east to the Kormakitis region in the west. Finally, the Circum Troodos zone, situated between the Troodos and Kyrenia mountains, is dominated by autochthonous sedimentary rocks of various origin. The Mesaoria Plain evolved mainly in the Quarternary and formed a land connection between the formerly separated mountain ranges [2].

The biogeography of Cyprus is shaped by its unique geographical position at the crossroads of Europe, Asia, and Africa. The island’s flora and fauna exhibit a pronounced Levantine character. At the same time, the strong European–Mediterranean influence and the island’s prolonged isolation have contributed significantly to the evolution of numerous endemic taxa. Consequently, Cyprus harbours one of the most diverse floras in the Mediterranean region relative to its size. A total of 1649 indigenous and 276 introduced taxa (species and subspecies) have been recorded to date [3]. Furthermore, both the flora and fauna of Cyprus are exceptionally rich in endemic species. Among plants, endemics account for approximately 7.4% of the native taxa. Several endemic vertebrates have also been recorded on the island, including the Cyprus Mouse (*Mus cypriacus*), the Cyprus Spiny Mouse (*Acomys nesiotes*), the Cyprus Warbler (*Curruca melanothorax*), the Cyprus Wheatear (*Oenanthe cypriaca*), and the Cyprus Scops Owl (*Otus cyprius*). Particularly noteworthy, however, is the remarkable number of endemic insect species found among the estimated 6000 insect taxa occurring on the island [4,5].

**Figure 1:**
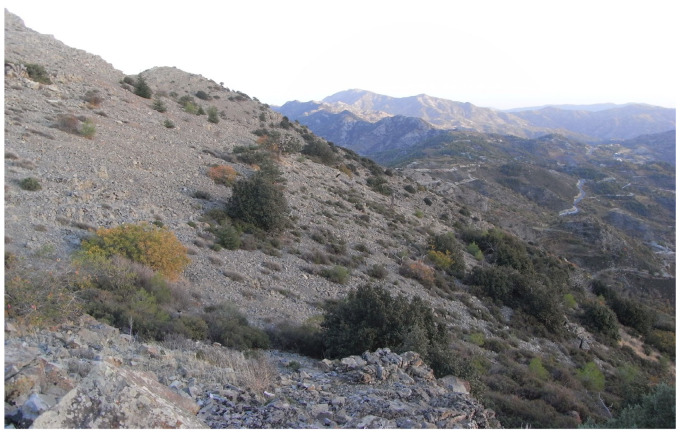
Eastern part of the Troodos Mountains (Adelfoi), characterized by extensive scree slopes (Photo E. Friedrich).

**Figure 2:**
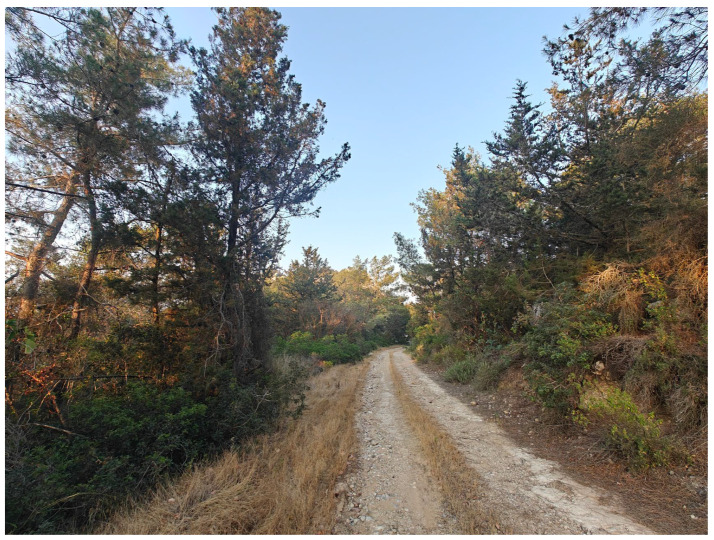
The formerly extensive pine and deciduous forests are today largely confined to the Troodos Mountains or, as shown here, to the Pentadaktylos Mountains (Photo P. Huemer).

**Figure 3:**
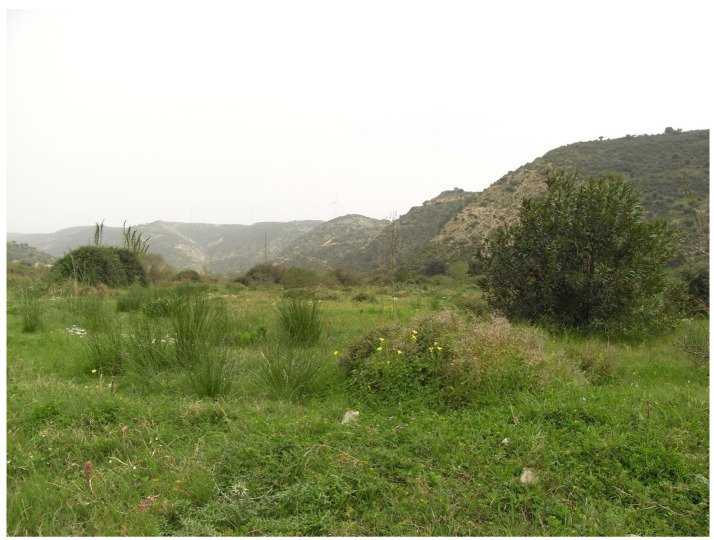
Wetland habitat near the Diarizos River, Nikokleia (Photo E. Friedrich).

**Figure 4:**
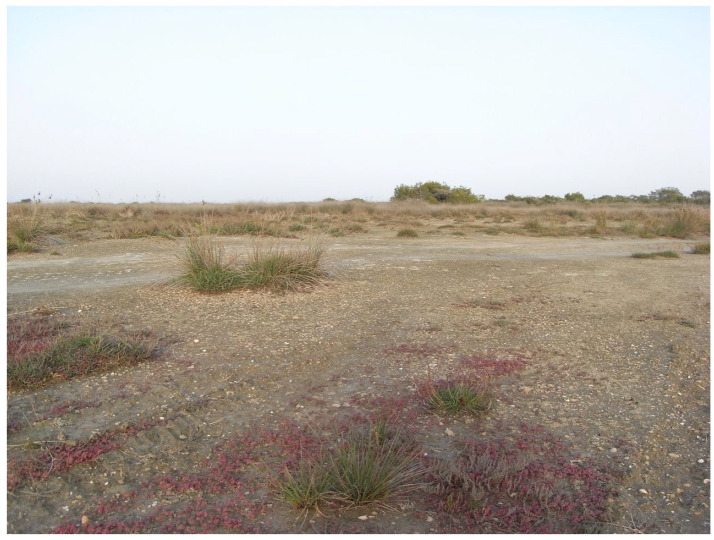
Salt marshes at Akrotiri, near Limassol (Photo E. Friedrich).

**Figure 5:**
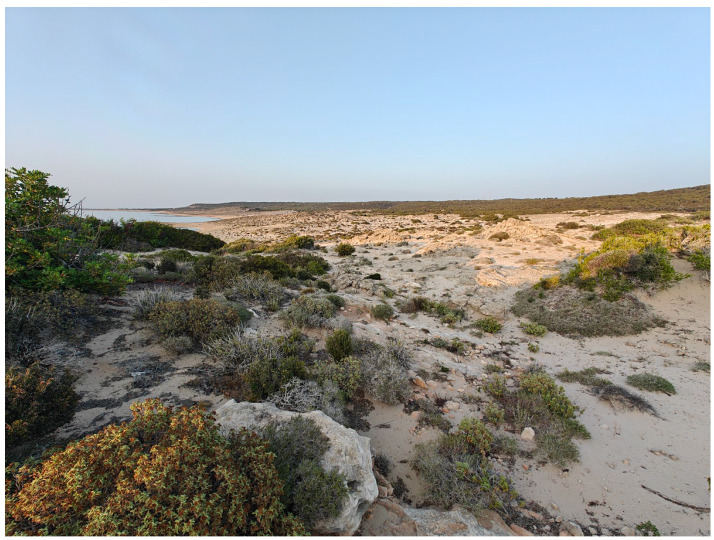
Well-preserved, unspoiled sand dunes are found particularly along the northern coast near Dipkarpaz (Photo P. Huemer).

The island’s diverse topography and high species richness are reflected in the remarkable variety of its habitat types. The riparian vegetation of oriental plane and alder, the endemic cedar forest, the cypress forests, and the relic forests of Cyprus oak are local formations. The extensive pine forests, sclerophyllous evergreen vegetation, high and low maquis, as well as garigue and phrygana, constitute the dominant woody vegetation types. Herbaceous vegetation mainly consists of grasslands, vegetation of sand dunes and cliffs, and plants typical of temporary ponds. Finally, in the high-altitude areas, scree slopes and rocky habitats with a xeromontane character are found, where vegetation is only sparsely developed (Figure 1, Figure 2, Figure 3, Figure 4 and Figure 5).

Cyprus is politically divided into several zones: the northern part (approximately 36% of the island) is administered by the Turkish Republic of Northern Cyprus, while the southern part (approximately 58%) is governed by the Republic of Cyprus. In addition, there are two British Sovereign Base Areas (approximately 2.8%) and a United Nations buffer zone separating the northern and southern parts of the island. However, this complex political situation is disregarded here, and the island is considered as a whole.

The lepidopteran fauna of Cyprus attracted the attention of scientists early on. The first sampling dates back to the mid-19th century, but remained rudimentary in scope, with only 91 species with few additions during the following decades [6,7]. More comprehensive studies were not carried out until after the British occupation of the island and the accompanying increased interest of researchers from the British Empire [8,9,10]. As in the case of Crete and several regions of the Balkan Peninsula, Hans Rebel (1861–1940), an Austrian lepidopterist and long-serving director of the Natural History Museum in Vienna, was the first to provide an overview of the Lepidoptera fauna of Cyprus in 1916 [11]. His study listed a total of only 166 species, which he estimated to represent approximately one-eighth of the island’s actual fauna. In the first and so far only comprehensive account of the Cypriot Lepidoptera, published in 1939, the number of recorded species had already increased to 482 [12]. Further significant advances were made especially by Edward Parr Wiltshire (1910–2004) and Hans Georg Amsel (1905–1999) in the mid-20th century [13,14], whereas later decades yielded relatively fewer, more targeted contributions, such as species descriptions and studies on economically relevant species [15,16,17,18,19]. It was not until the 1980s that the lepidopteran fauna of Cyprus was again surveyed more systematically. Here it is primarily owing to the work of the Austrian lepidopterists Ernst Arenberger (1933–2020) and Josef Wimmer (1935–2016) that the remarkable diversity of microlepidoptera in Cyprus was investigated in depth and comprehensively documented for the first time [20,21,22,23]. In particular, Arenberger’s first comprehensive work in 1994 more than doubled the species number in these families, reaching a total of 461 species [20]. Further important faunistic additions to microlepidoptera were made by authors of this study through their own sampling [24,25,26]. Numerous faunistic and taxonomically relevant studies on macrolepidoptera were published, particularly toward the end of the 20th century [27,28,29,30,31,32,33]. A summary of the knowledge on most diverse families of larger moths and butterflies was provided by the German lepidopterists Fischer and Lewandowski [34,35,36], as well as in a comprehensive monograph on the butterflies of Cyprus by Manil [37]. However, since Rebel’s time, no comprehensive inventory of the total Lepidoptera fauna of Cyprus has been attempted. The now-defunct *Fauna Europaea* database, which is still fully accessible through the PESI platform and integrated into Lepiforum, provided a complete species list, but frequently lacked references and contained numerous errors [38,39]. An effort by the renowned Hungarian lepidopterist László Gozmány (1921–2006) to compile a comprehensive inventory of the Lepidoptera of Greece and Cyprus was discontinued after the first volume due to his death [40]. The issue of insufficient faunistic and taxonomic data therefore remains highly relevant. This situation is further exacerbated by the fact that the island’s fauna has so far been studied almost exclusively using classical, mostly morphological methods, which in many cases do not allow for reliable species delimitation. A more integrative approach incorporating molecular data has been applied only in a few exceptional cases [41,42,43,44,45,46].

To address these substantial deficiencies, a joint research project initiated by Near East University (ÖÖ) and the Tyrolean State Museums (PH) aimed, for the first time, to genetically document as many species as possible using DNA barcoding [26]. The inclusion of extensive recent collections by several colleagues, digital observation data, and the critical reassessment of earlier publications now makes it possible—despite remaining gaps and unresolved taxonomic problems—to compile, for the first time, a well-founded inventory of the island’s Lepidoptera fauna.

## 2. Materials and Methods

### 2.1. Field Surveys

**Figure 6:**
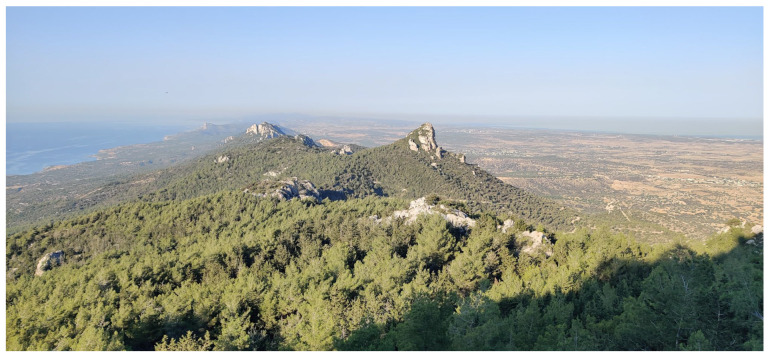
Recent surveys in Northern Cyprus focused primarily on the Pentadaktylos Mountains (Photo P. Huemer).

**Figure 7:**
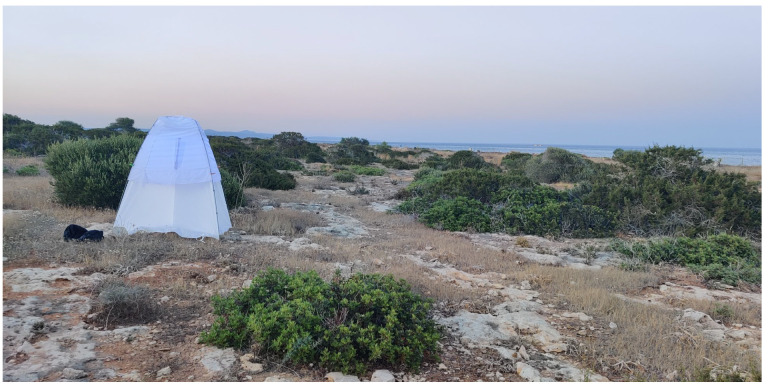
One of the main methods for sampling nocturnal moths was the use of various light sources (Photo P. Huemer).

Field surveys were primarily based on intensive fieldwork conducted by the authors PH, IB, and JJ in Cyprus. In north Cyprus, comprehensive sampling was carried out by PH with support from ÖÖ during five excursions between autumn 2023 and 2025, with the primary objective of obtaining genetic sequences for all accessible species, except for the Papilionoidea, which have already been extensively studied on the island (Figure 6 and Figure 7). In contrast, the Lepidoptera of the southern part of the island were surveyed over more than two decades by IB and JJ, supported by the sampling efforts of numerous other lepidopterists. These surveys were performed in an unsystematic manner, i.e., without a clearly defined sampling protocol. Furthermore, only a limited subset of species has been genetically analyzed to date, and several additional unpublished faunistic records are therefore provided in Table S1 as a reference for future studies. Nevertheless, the overarching goal was to achieve the most complete possible documentation of the local fauna, albeit without a specific emphasis on subsequent genetic analyses.

To ensure a representative inventory, the main habitats of Cyprus were sampled throughout all seasons. Lepidoptera-specific sampling methods were applied as required. Surveys of the particularly diverse nocturnal families were conducted primarily using various light sources and, less frequently, alternative methods such as bait traps or vegetation beating. Diurnal species and preimaginal stages were sampled through visual searches, netting, or pheromone traps.

Specimens were collected as needed, treated with acetic ether or comparable narcotizing agents, and, in most cases, immediately pinned, spread, and dried in the field (PH), or pinned and later softened and properly mounted by the other authors. Preimaginal stages were reared in the authors’ laboratories, and upon adult emergence, specimens were treated in the same manner.

### 2.2. DNA Barcoding

Tissue samples (dried legs) from 1349 specimens from the Tyrolean State Museum (TLMF) and provisionally identified to the morphospecies level were prepared according to standard protocols to obtain DNA barcode sequences of the mitochondrial COI gene (cytochrome c oxidase I). The material was processed at the Centre for Biodiversity Genomics (University of Guelph, Guelph, ON, Canada) using a standard high-throughput workflow [47]. The remaining 689 specimens, most of which were already publicly available in the Barcode of Life Data System (BOLD) [48], originated from several additional collections, primarily the Zoologische Staatssammlung München (ZSM; 154 specimens), the Finnish Museum of Natural History (FMNH, Helsinki; 87 specimens), the Zoological Museum of the University of Oulu (ZMUO; 87 specimens), and especially leafminers were sequenced at Naturalis Biodiversity Center, Leiden (RMNH, 35 specimens). Part of this material was processed within the framework of the Biodiversity Genomics Europe (BGE) project using the corresponding protocol as described in [49].

Details, including complete voucher information and specimen images, are available in the public dataset “Barcoding Lepidoptera of Cyprus” (https://dx.doi.org/10.5883/DS-LEPICYPR) within the Barcode of Life Data System (BOLD). With the exception of ten public sequences, all relevant data are included in this resource.

All sequences were assigned to Barcode Index Numbers (BINs), an algorithm-based approach to delineate operational taxonomic units that provide a good proxy for species—which were automatically calculated for records in BOLD that were compliant with the DNA barcode standard [50]. A few BINs included specimens belonging to more than one taxon because of BIN sharing, misidentifications, or contaminations. Identification was based on external morphology and, in critical cases, on genitalia morphology. In the case of BINs attributed to a single Linnean name, these were accepted as correct although misidentifications cannot be ruled out.

**Figure 8:**
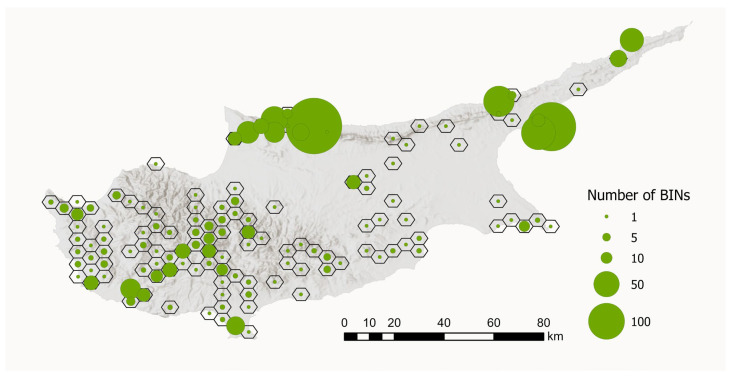
Number of BINs per sampling region in Cyprus. Sampling sites that were close together were aggregated within hexagons with a diameter of 5 km (*n* = 130). The size of the circles indicates the number of BINs. Map data source: ESRI, USGS, Statistical Service of Cyprus.

To visualise the geographical distribution of the BIN samples, we created a map showing the number of BINs per sampling site. To improve visualisation and account for sampling sites that were very close together, we aggregated sites within hexagons with a diameter of 5 km. This aggregation resulted in 130 hexagons. The number of unique BINs per 5 km hexagon ranged from 1 to 239 (see Figure 8).

### 2.3. Checklist

The checklist of the Lepidoptera of Cyprus is primarily based on the national fauna database provided by Lepiforum, which already incorporates a large proportion of published species records. The taxonomic arrangement and nomenclature follow this online resource as well, except for a few disputed genera and subgenera of the Noctuidae. Synonyms and subspecies were deliberately excluded.

By critically reviewing additional literature and data sources not previously included in Lepiforum, several further species were added to the checklist. Moreover, a substantial number of species, confirmed morphologically and/or genetically from our own sampling, were recorded for the first time in Cyprus and incorporated into the faunal inventory. In addition, a large number of previously published species were sampled in this study and the corresponding detailed data are presented here. The checklist additionally includes information on BINs and the occurrence status of individual species in Cyprus. Furthermore, relevant bibliographic references are provided which, with the exception of the endemic taxa, are largely based on comprehensive summary works.

Taxa that can currently be identified only to the genus level based on genetic data and/or morphological analyses are not included in the main checklist. In addition, misidentifications resulting from various errors, as well as highly questionable species, were excluded from the island’s fauna.

## 3. Results

### 3.1. DNA Barcodes—General Overview

The analysis of tissue samples from 2042 specimens yielded 1859 DNA barcode sequences, representing approximately 50% of the Lepidoptera fauna of Cyprus. Full DNA barcodes (658 bp) without stop codons were recovered for 1455 specimens, while only 31 specimens had a sequence shorter than 500 bp. Finally, sequencing failed for 174 specimens. Sequences clustered into 701 BINs (Figure 8).

The historically divergent sampling strategies and the associated processing of specimens resulted in a pronounced bias in the dataset, with a clear predominance of both samples and BINs from north Cyprus (Figure 8). DNA barcodes are available for nearly all recorded species from this region, whereas data from the southern part of the island is fragmentary.

### 3.2. BINs Attributed to Linnaean Names

**Figure 9:**
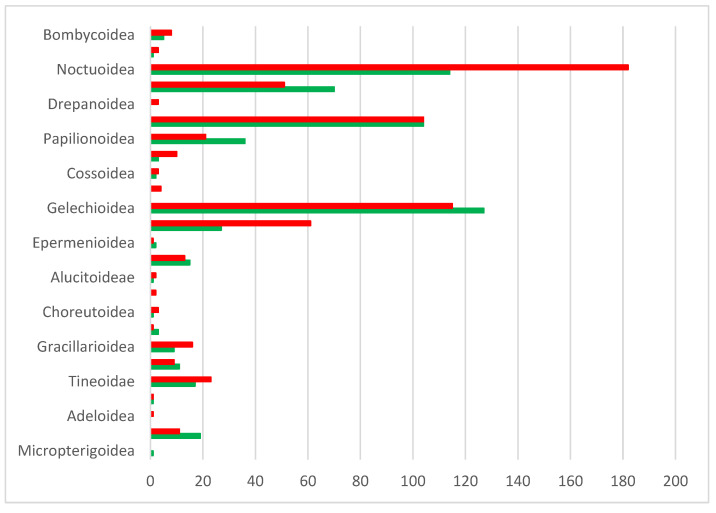
Number of Linnaean species per superfamily in Cyprus (systematic order), with BIN coverage (green) and without BIN (red). BINs without a species assignment were excluded.

BOLD analytical tools assigned species-level identifications to 596 BINs, corresponding to 580 Linnean names (Table S1). Sequences for five additional species either lacked a BIN or had a BIN pending. The genetic coverage of the Linnaean species inventory based on BINs is approximately 48% overall but varies considerably among the major systematic groups (superfamilies). Eleven of 25 superfamilies have barcode coverage for more than half their component species, but two species-rich taxa (Noctuoidea, Tortricoidea) fail to meet this mark. A similar situation applies—despite a comparatively higher BIN coverage—to the diverse superfamilies Pyraloidea, Gelechioidea, and Geometroidea (see Figure 9).

Fourteen species were assigned to two BINs, including *Ateliotum arenbergeri*, *Tecmerium perplexum*, *Cydia fagiglandana*, *Agdistis tamaricis*, *Stenoptilia aridus*, *Maniola cypricola*, and *Hipparchia syriaca*, while *Acalypris pistaciae* and *Symmoca salem* were assigned to three BINs. Such cases may reflect deep intraspecific variability in the DNA barcode, as observed in *Cydia fagiglandana* and *Tecmerium perplexum*, species for which extensive genital morphological studies revealed no evidence of overlooked diversity. In other cases, introgression may explain strongly divergent genetic clusters. For example, one specimen of *Hipparchia syriaca* clustered with *Hipparchia alcyone*, a species not yet recorded anywhere in Southeastern Europe or the Near East. Genital morphology, however, unequivocally confirms the specimen as *H. syriaca*. It is also possible that cryptic diversity exists in some taxa with deep barcode divergence, but these taxa await investigation. In contrast, only four species in Cyprus— all belonging to the genus *Agonopterix*—share the same BIN.

A total of 109 species exhibit a unique BIN within the BOLD database, including two pairs of variable species (four species in total) represented by two or three BINs in Cyprus. In most cases, these endemic BIN members correspond to true island endemics. Other, more widely distributed species show only relatively minor divergence from their Linnaean counterparts—typically around 2%—despite being placed in different BINs are treated as one species when no clear morphological differences are apparent. Examples include *Metzneria artificella*, *Scrobipalpa bigoti*, *Mirificarma eburnella*, *Pyralis kacheticalis*, and *Capperia celeusi*. Finally, the group of endemic BINs also includes several highly divergent subspecies.

Many species cluster into multiple BINs across their European range, but the Cypriot fauna often exhibits clear affinities with extra-European clusters, primarily from the Near East or the Levantine region. Notable examples include *Zeuzera pyrina*, *Scythris mus*, *Pararge aegeria*, *Amephana dalmatica*, and *Amphipyra micans*. All these taxa require a comprehensive morphological analysis and comparisons with potentially relevant Linnaean species, ideally based on a study of the type material. While minor genetic divergences are temporarily attributed to the nominate species, strongly divergent clusters (>3%) are provisionally assigned to currently unidentified species (Table S2). An exception is *Pachythelia villosella quadratica* de Freina, 1983, which, with a genetic divergence of more than 5%, is likely to represent a distinct species but is still listed under the name of the nominotypical subspecies; and further three species of Nepticulidae that are highly variable in barcodes, but morphologically inseparable from other populations.

### 3.3. New Faunistic Records

#### 3.3.1. New Records with Accompanying DNA Barcodes

In total, 57 Lepidoptera species belonging to 21 families were newly recorded for Cyprus through this study, based in part on their DNA barcodes (Table 1). These additions expand the known faunal inventory and provide a genetic reference for future taxonomic and biodiversity research. Detailed collection information and sequences are available in the dataset “Barcoding Lepidoptera of Cyprus” (https://dx.doi.org/10.5883/DS-LEPICYPR; see Table S3). For several of these species, additional unsequenced voucher material is preserved in the collections of TLMF, the Cyprus Herbarium and Natural History Museum, Nicosia (CHNHM), JJ, and IB. In some cases, photographic records are also available on online platforms.

The list includes five noteworthy new records for Europe, namely *Alloclita deprinsi*, *Cochylimorpha additana*, *Cydia alienana*, *Ephestia abnormalella*, and *Hypsotropa paucipunctella*.

#### 3.3.2. New Records Based on Morphology

A total of 62 species representing 20 families were newly recorded from Cyprus through morphological examination. Most identifications were confirmed from voucher specimens based on both external morphology and genitalia dissections. In contrast, five species—*Bifasciodes leucomelanella*, *Watsonalla uncinula*, *Bryophilopsis roederi*, *Xanthodes albago*, and *Aphomia sabella*—were identified solely from live photographs posted on online forums, without voucher specimens. Fife species—*Bryophilopsis roederi*, *Cochylimorpha diana*, *Pammene avetianae*, *Pammene nannodes*, and *Dysauxes parvigutta*—constitute first records for Europe (Table 2; label data in Table S4).

### 3.4. Unidentified Species—Potential Cryptic Diversity

#### 3.4.1. Unidentfied BINs

A total of 105 Barcode Index Numbers (BINs) could not be assigned to any Linnaean species, even after preliminary morphological analyses, due to the absence of reference sequences in BOLD. Of these, 68 BINs are currently known exclusively from Cyprus, while the remaining BINs were already on BOLD but not classified at the species level. While two tineid BINs could only be identified to the family level, the remaining BINs were assigned to a genus (Table S2). Images of the relevant species can be found at https://dx.doi.org/10.5883/DS-LEPICYPR.

**Figure 10:**
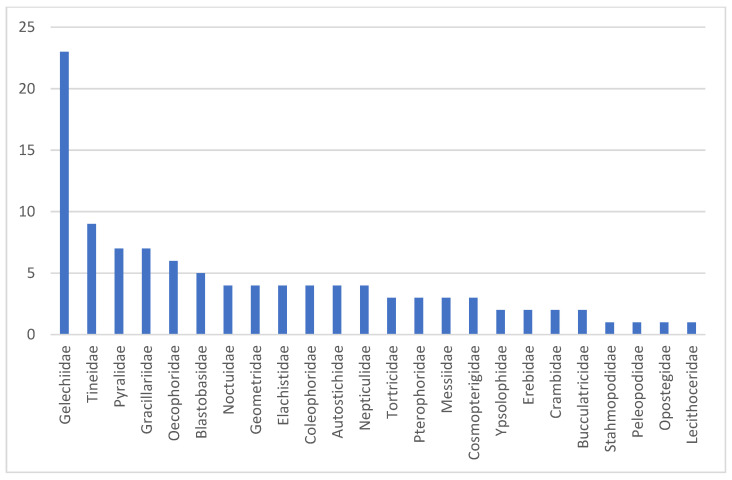
Number of unassigned BINs per family.

The BINs that cannot be assigned to species level are distributed very unevenly across 24 families (Figure 10). The number of unidentified BINs is, by far, the highest in Gelechiidae, but also other microlepidopteran families (Tineidae, Pyralidae, Oecophoridae, Gracillariidae) include multiple unknowns (Figure 10). In some families, such as the Tineidae, the currently limited coverage in BOLD constrains species assignment, whereas this is far less the case in Gelechiidae, suggesting the presence of several undescribed species in Cyprus [51]. The genetic distances to the nearest neighbor in BOLD also vary greatly, ranging from only 1.12% to 11.24%. The particularly low divergences, however, refer to unnamed reference sequences in BOLD. For 60 BINs, the distance to the nearest neighbor exceeds 3%.

The unassigned BINs belong either to insufficiently revised species complexes or to taxa lacking adequate documentation, for instance due to missing illustrations or superficial original descriptions. Further integrative taxonomic studies—combining morphological, genetic, and ecological data—are essential to resolve these cases. The following examples illustrate the complexity and taxonomic challenges associated with some of these unassigned taxa.

*Oegoconia* sp. (Autostichidae)

The taxon identified as *O. deauratella* is represented in Cyprus by three endemic Barcode Index Numbers (BOLD:ADF0714, BOLD:AGP5600, and BOLD:AHC3739), which show very low intraspecific variation. In contrast, these BINs exhibit substantial genetic divergence (>5%) from *O. deauratella* sensu stricto [24]. This evidence suggests the presence of an undescribed, genetically variable species endemic to Cyprus.

*Lecithocera* sp. (Lecithoceridae)

Barton reported *L. anatolica*, originally described from Türkiye, as a new record for Cyprus [25]. His identification was based on a previously undescribed female of the species, which was determined using comparative material of both sexes from Israel [52]. However, the species assignment remains uncertain due to the absence of a direct comparison with the type material. Examination of the male genitalia of an independently collected specimen by JJ did not allow confident identification based on the schematic illustrations provided by Gozmány [53].

*Isophrictis* spp. (Gelechiidae)

The genus *Isophrictis* is in urgent need of taxonomic revision. The four BINs recorded from Cyprus exhibit only minor genetic divergence (<2%) from their nearest neighbours. However, with one exception, they cannot currently be assigned to a species with confidence. Two BINs (BOLD:AEI4278 and BOLD:AEI4279) are so far known exclusively from Cyprus. The latter is tentatively attributed to *I. kefersteiniellus* based on its low genetic divergence (1.24%) and phenotypic congruence with that species.

#### 3.4.2. Morphology-Based Unidentified Species

A total of 35 taxa were classified solely on the basis of morphological characteristics but have not yet been identified to species level. Table S5 provides an overview of these taxa together with detailed information on the respective collection circumstances.

### 3.5. Critical Review of Doubtful or Incorrect Records from Cyprus

Over the long history of studies on the Lepidoptera of Cyprus, many species have been reported whose occurrence on the island is either highly questionable or can be excluded altogether for various reasons. As early as the mid-19th century, Rebel listed numerous doubtful records that have never been reconfirmed [11,12].

Most of these erroneous reports result from the following causes:

(a) misidentifications of voucher specimens or, more recently, photographs;
(b) changes in species concepts;
(c) unresolved genetic divergences indicating overlooked cryptic diversity;
(d) technical errors (e.g., incorrect data tables or specimen mislabelling).

Although examination of the original material was only possible in a few cases, we excluded the doubtful or clearly incorrect records (Table S6) from the checklist—even in the absence of a full revision of the largely inaccessible historical collections. Species records were critically evaluated, particularly when their known distribution or morphological similarity to other taxa made their presence in Cyprus implausible. Most of these species were listed in *Fauna Europaea* without citation of a primary source, and many of the erroneous records summarized here have already been addressed in previous publications. As a result of this critical assessment, 158 species were ultimately excluded from the faunal list (Table S6). The reasons for the exclusion of species without a current identity assignment (“?” in Table S6) can be found in detail in Lepiforum (Faunistics) and are not repeated here [39].

In addition, several photographic records from iNaturalist [54] were also problematic, particularly when secondarily incorporated—albeit with uncertainty tags—*into* Lepiforum. Ultimately, several purported new records, including *Micropterix aruncella*, *Stemmatophora combustalis* (confirmed, however, by material ex coll. Junnilainen), and *Eilema caniola*, were corrected on iNaturalist and no longer appear under the originally suggested names. However, since these were user-generated identification proposals rather than formal scientific publications, we have refrained from listing these temporary misidentifications in detail.

### 3.6. Revised and Updated Checklist

The revised checklist of the Lepidoptera of Cyprus now includes 1213 Linnaean species belonging to 65 families (Table S1). It includes about 1150 native species with established, self-sustaining populations. In contrast, at least 37 alien species are recorded, including 16 confirmed alien species and 21 cryptogenic species referring to taxa of uncertain origin [55,56,57], Table S1. Finally, at least 30 species are categorized as presumably non-resident migratory species, but temporary establishment may be possible for some of them. Conversely, for several native species, sporadic or even regular immigration seems likely. Together with the yet unidentified species, the lepidopteran fauna of Cyprus is likely to include well over 1300 species.

**Figure 11:**
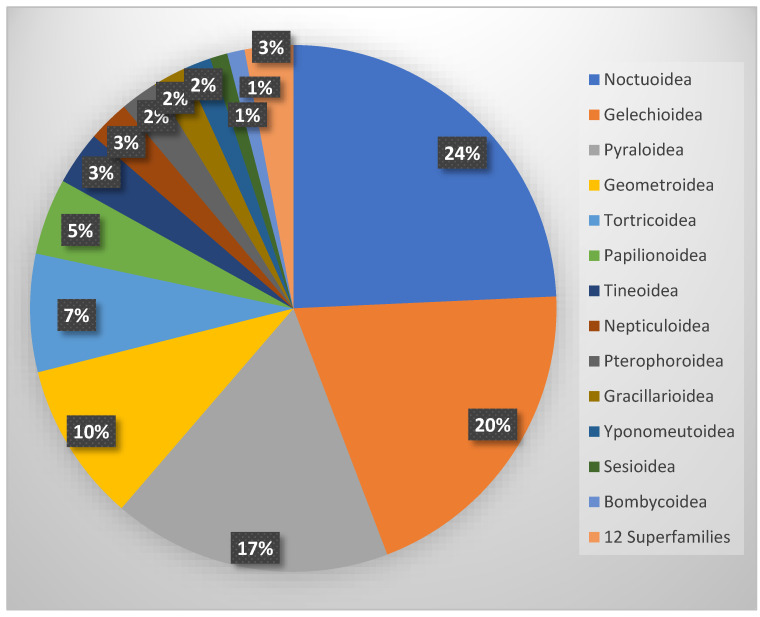
Proportion of superfamilies in the total Lepidoptera fauna of Cyprus.

The systematic analysis of the faunal composition reveals a clear dominance of a few highly diverse superfamilies. Of the 25 superfamilies present, the Noctuoidea (295 spp.), Gelechioidea (242 spp.), Pyraloidea (208 spp.), and Geometroidea (119 spp.) are the most species-rich, each comprising more than 100 species, whereas 12 superfamilies are represented by five or fewer species (Figure 11).

### 3.7. Insular Endemism

**Figure 12:**
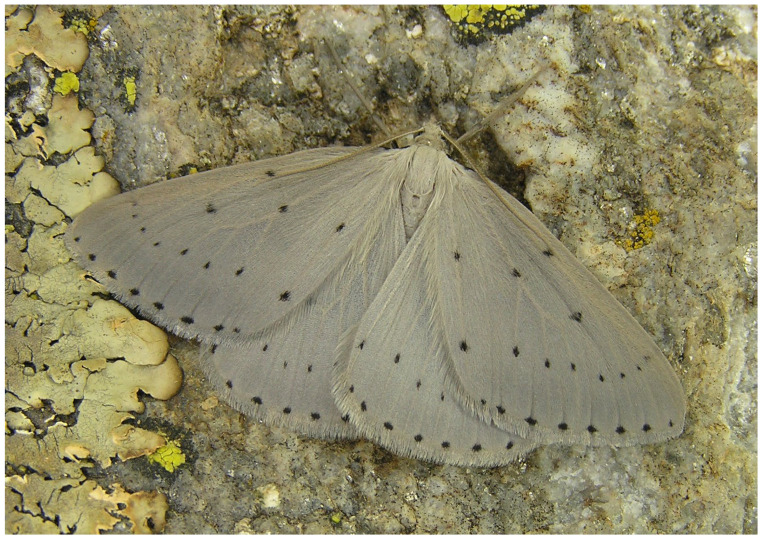
The endemic *Orthostixis cinerea*, first described by Rebel in 1916 (Photo E. Friedrich).

**Figure 13:**
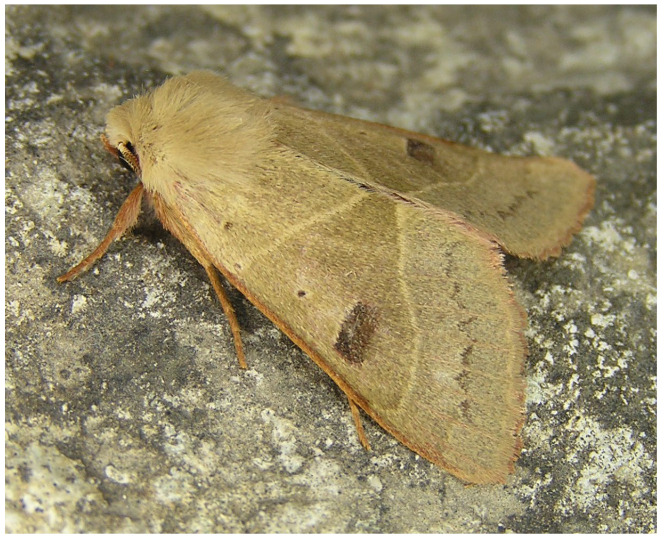
*Pseudenargia troodosi*, one of the several island-endemic species emerging only in autumn (Photo E. Friedrich).

Cyprus is currently thought to host 55 endemic species (Table S1), but some of them, especially those that are inconspicuous, may well also occur on the mainland, particularly in Turkey and the Levant countries. Conversely, island endemism appears highly probable for numerous phenotypically distinct species, especially butterflies and macro-moths (Macroheterocera) (Figure 12 and Figure 13). Notable examples include *Glaucopsyche paphos*, *Hipparchia cypriensis*, *Orthostixis cinerea, Nychiodes aphrodite*, *Pseudoterpna rectistrigaria*, *Dichagyris endemica*, *Perigrapha wimmeri*, *Pseudenargia troodosi* and *Ammoconia aholai*. Interestingly, most of the endemic Noctuoidea are active in autumn or winter and were therefore only discovered and described recently, most within the past 20–30 years.

**Figure 14:**
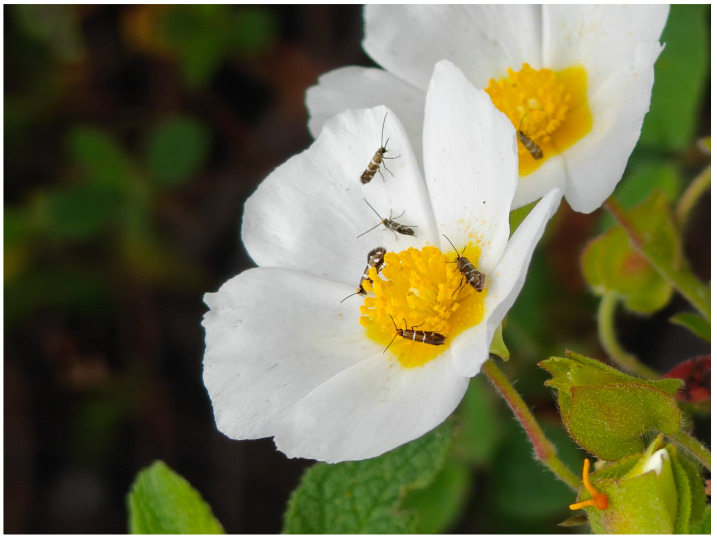
*Micropterix cypriensis* is an unmistakable endemic species of Cyprus (Photo P. Huemer).

Endemism is also highly likely for several more conspicuous micro-moth species, such as *Micropterix cypriensis* and *Batia hilszczanskii* (Figure 14). The same applies to species with trophic associations to recognized endemic plants, such as *Phyllonorycter troodi*, which is associated with *Quercus alnifolia*.

Genetic data are lacking for many of the numerous endemic subspecies described from Cyprus that may represent valid species. Preliminary molecular results, together with the observed divergences from the nominate subspecies, indicate the need for necessary taxonomic re-evaluations. Examples of such unresolved genetic distances include *Synanthedon myopaeformis luctuosa* (4.6%), *Colotois pennaria paupera* (2.94%), *Autophila anaphanes cypriaca* (1.8%), and *Allophyes asiatica cypriaca* (2.21%).

## 4. Discussion

Cyprus has been the subject of lepidopterological investigation and sampling for approximately 170 years, although with varying intensity. Despite this long research history, our study reveals a striking lack of comprehensive and up-to-date faunistic syntheses, as well as the absence of a critical re-evaluation of previously published records. Moreover, genetic analyses—particularly DNA barcoding—have never before been systematically applied to the island’s lepidopteran fauna.

As a result of our integrative approach, a considerable number of species had to be removed from the local faunal list due to insufficient or demonstrably incorrect documentation. At the same time, the revised checklist was substantially enriched by more than 100 newly confirmed Linnean species, verified through genetic and/or morphological evidence. Of these newly confirmed species, 10 also represent first records for Europe. Nevertheless, the current total of approximately 1200 species is likely a considerable underestimate of the actual lepidopteran diversity in Cyprus. More than 100 unidentified Barcode Index Numbers (BINs), along with several morphologically recorded but taxonomically unresolved taxa, point to substantial gaps that remain in the faunal inventory.

The genetic data generated in this study, currently covering about half of the known species, provides an essential tool for future taxonomic, phylogeographic, and biogeographical research. The resulting barcode library shows close correspondence with recently published data for Crete—a comparable island in terms of size, topographic diversity, and lepidopterological history [49]. While Crete hosts 1230 Linnaean species (of which 724 have been genetically sequenced), 125 species were confirmed there for the first time through DNA barcoding. In Cyprus, the corresponding figures are 1213 species, with 57 new records confirmed genetically and 62 based solely on morphology. Similarly, the number of taxa excluded from the Cypriot faunal list (158 spp.) is comparable to the 212 species removed from the Cretan checklist. Of particular note is the unexpectedly high number of currently unidentified sequence clusters on both islands—105 in Cyprus and 112 in Crete—underscoring the urgent need for further integrative taxonomic revision.

**Figure 15:**
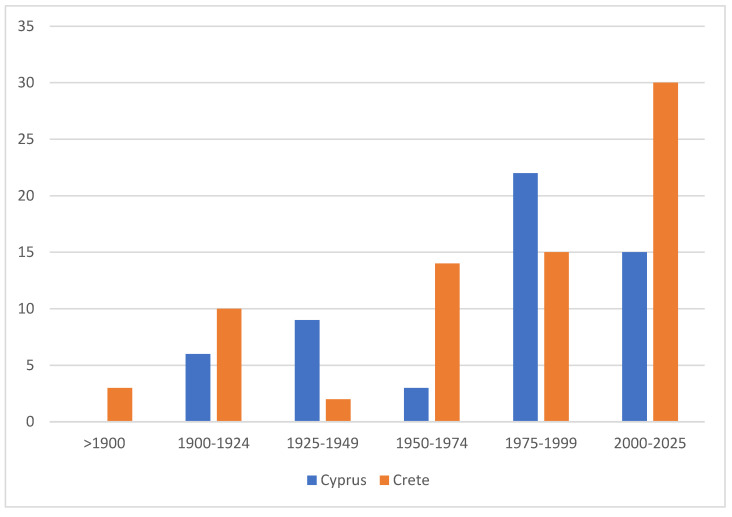
Number of endemic Lepidoptera species described in different time periods in Cyprus and Crete. Data indicate the cumulative number of species recognized as endemic during each period, highlighting the continuing rate of discovery in both faunas.

The degree of endemism is also relatively similar between the two islands, although Crete harbors a slightly higher number and proportion of endemic taxa, with 75 species currently recognized compared to 55 in Cyprus (Figure 15). The somewhat higher degree of endemism in Crete is presumably attributable to the island’s longer isolation, but, notably, not to the higher mountain ranges, which, based on currently available data, do not seem to harbor any distinct endemic species. The considerable proportion of endemic species is the result of the long-term isolation of both islands and clearly exceeds that of the mainland-proximate island of Sicily [58]. In contrast, the significance of endemism is even greater on remote islands such as Madeira, where 57 of the 331 recorded species—approximately 17% of the total fauna—are endemic [59].

Notably, approximately two-thirds of the Cypriot endemics have been described since 1975, including 15 species that were added after 2000. Although the occurrence of some taxa outside Cyprus would not be unexpected, approximately 5% of the island’s Lepidoptera fauna can nonetheless be considered endemic. Remarkably, even in Rebel’s 1916 treatment, about 6% of the then-listed 166 Cypriot species were already identified as endemics—a proportion that has remained largely stable, despite the seven-fold increase in the number of known species since that time. While Cyprus appears somewhat less rich in endemic taxa than Crete, a precise comparison remains difficult due to numerous unresolved taxonomic issues and the still incomplete molecular coverage of both faunas. The somewhat higher degree of endemism in Crete is presumably attributable to the island’s longer isolation and its highly structured landscape with numerous canyon habitats, but notably not to its higher limestone-dominated mountain ranges, which, based on currently available data, do not appear to harbor any distinct endemic species.

Further discoveries of Cypriot endemics are particularly likely within the 71 unidentified genetic clusters currently known only from the island. However, even seemingly well-studied groups and taxa previously considered unambiguously identified may reveal additional diversity. A notable example is the recently described *Episema amettai* [60].

Ultimately, a more reliable assessment of the Lepidoptera of Cyprus will only be possible through further integrative studies combining genetic and morphological approaches encompassing the entire island fauna. Even in relatively well-documented families such as Noctuidae and Erebidae, a surprisingly large number of species complexes require taxonomic revision, despite the apparent availability of well-established identification literature, at least for the European fauna [61,62,63,64,65,66,67]. Accordingly, we plan to carry out further sequencing of as-yet unstudied species, along with comprehensive taxonomic revisions. We emphasize that achieving comprehensive genetic coverage for European and Western Palaearctic Lepidoptera represents a fundamental step toward closing the existing sequencing gaps and advancing our understanding of these taxa [68].

## 5. Conclusions

Genetically based species inventories are a crucial prerequisite for high-quality biodiversity assessments. However, such well-founded checklists remain the exception even in Europe and currently exist for Lepidoptera only in Finland [69]. In the Mediterranean region in particular, historically developed, purely morphology-based inventories often reach their limits due to insufficient taxonomic knowledge, resulting in frequent misclassifications in faunal lists [70].

With the increasing coverage of European species in the Barcode of Life Data Systems (BOLD), the establishment of robust regional inventories based on genetic reference sequences has become feasible as demonstrated for Crete [49] and now for Cyprus.

The still considerable number of genetically validated but unclassified taxa—especially endemic clusters—revealed here highlights persistent gaps in the European DNA barcode library. These gaps must be closed through targeted, integrative taxonomic studies to ultimately achieve complete and reliable faunistic inventories.

## Figures and Tables

**Table 1:** A total of 57 species newly recorded for the fauna of Cyprus largely based on DNA barcodes.

Taxon	Family
*Alucita zonodactyla* Zeller, 1847	Alucitidae
*Coleophora variicornis* Toll, 1952	Coleophoridae
*Alloclita deprinsi* Koster & Sinev, 2003	Cosmopterigidae
*Anatrachyntis badia* (Hodges, 1962)	Cosmopterigidae
*Limnaecia phragmitella* Stainton, 1851	Cosmopterigidae
*Dolicharthria metasialis* (Rebel, 1916)	Crambidae
*Glaucocharis euchromiella* (Ragonot, 1895)	Crambidae
*Agonopterix rotundella* (Douglas, 1846)	Depressariidae
*Depressaria bantiella* (Rocci, 1934)	Depressariidae
*Klimeschia lutumella* (Amsel, 1938)	Douglasiidae
*Elachista griseella* (Duponchel, 1843)	Elachistidae
*Eilema costalis* (Zeller, 1847)	Erebidae
*Epermenia aequidentellus* (Hofmann, 1867)	Epermeniidae
*Caryocolum proxima* (Haworth, 1828)	Gelechiidae
*Dichomeris helianthemi* (Walsingham, 1903)	Gelechiidae
*Gelechia obscuripennis* (Frey, 1880)	Gelechiidae
*Helcystogramma lutatella* (Herrich-Schäffer, 1854)	Gelechiidae
*Isophrictis kefersteiniellus* (Zeller, 1850)	Gelechiidae
*Parastenolechia nigrinotella* (Zeller, 1847)	Gelechiidae
*Pexicopia malvella* (Hübner, 1805)	Gelechiidae
*Scrobipalpa spergulariella* (Chrétien, 1910)	Gelechiidae
*Stenolechiodes macrolepiellus* Huemer & Karsholt, 1999	Gelechiidae
*Teleiodes luculella* (Hübner, 1813)	Gelechiidae
*Telphusa cistiflorella* (Constant, 1889)	Gelechiidae
*Acanthovalva inconspicuaria* (Hübner, 1819)	Geometridae
*Eupithecia irriguata* (Hübner, 1813)	Geometridae
*Eupithecia unedonata* Mabille, 1868	Geometridae
*Ectoedemia caradjai* (Groschke, 1944)	Nepticulidae
*Ectoedemia contorta* van Nieukerken, 1985	Nepticulidae
*Zimmermannia monemvasiae* (van Nieukerken, 1985)	Nepticulidae
*Bryophila felina* (Eversmann, 1852)	Noctuidae
*Nycteola asiatica* (Krulikovsky, 1904)	Nolidae
*Opostega spatulella* Herrich-Schäffer, 1855	Opostegidae
*Capperia loranus* (Fuchs, 1895)	Pterophoridae
*Hellinsia coniodactylus* (Staudinger, 1859)	Pterophoridae
*Achroia grisella* (Fabricius, 1794)	Pyralidae
*Acrobasis obtusella* (Hübner, 1796)	Pyralidae
*Acrobasis xanthogramma* (Staudinger, 1870)	Pyralidae
*Aglossa signicostalis* Staudinger, 1870	Pyralidae
*Delplanqueia inscriptella* (Dupopnchel, 1836)	Pyralidae
*Dioryctria pineae* (Staudinger, 1859)	Pyralidae
*Ephestia abnormalella* Ragonot, 1887	Pyralidae
*Hypsotropa paucipunctella* Ragonot, 1895	Pyralidae
*Pempelia albariella* Zeller, 1839	Pyralidae
*Peoria cremoricosta* (Ragonot, 1895)	Pyralidae
*Episcythris triangulella* (Ragonot, 1874)	Scythrididae
*Elatobia bugrai* Koçak, 1981	Tineidae
*Bactra bactrana* (Kennel, 1901)	Tortricidae
*Clepsis trivia* (Meyrick, 1913)	Tortricidae
*Cnephasia cupressivorana* (Staudinger, 1870)	Tortricidae
*Cochylimorpha additana* (Kennel, 1901)	Tortricidae
*Cydia alienana* (Caradja, 1916)	Tortricidae
*Endothenia ustulana* (Haworth, 1811)	Tortricidae
*Lobesia artemisiana* (Zeller, 1847)	Tortricidae
*Lobesia limoniana* (Millière, 1860)	Tortricidae
*Lobesia porrectana* (Zeller, 1847)	Tortricidae
*Zelleria hepariella* Stainton, 1849	Yponomeutidae

**Table 2:** A total of 62 new faunistic records from Cyprus, based solely on morphological evidence. Species identifications derived from online forums, based exclusively on photographs and lacking voucher specimens, are marked with an asterisk (*).

Species	Family
*Bedellia ehikella* Szőcs, 1967	Bedelliidae
*Coleophora ochrea* (Haworth, 1828)	Coleophoridae
*Coleophora pennella* (Denis & Schiffermüller, 1775)	Coleophoridae
*Bifascioides leucomelanella* (Rebel, 1917) *	Cosmopterigidae
*Cosmopterix pulchrimella* Chambers, 1875	Cosmopterigidae
*Dolicharthria stigmosalis* (Herrich-Schäffer, 1848)	Crambidae
*Euchromius vinculellus* (Zeller, 1847)	Crambidae
*Eudonia speideli* Leraut, 1982	Crambidae
*Evergestis frumentalis* (Linnaeus, 1761)	Crambidae
*Patania balteata* (Fabricius, 1798)	Crambidae
*Pyrausta purpuralis* (Linnaeus, 1758)	Crambidae
*Agonopterix cnicella* (Treitschke, 1832)	Depressariidae
*Agonopterix subpropinquella* (Stainton, 1849)	Depressariidae
*Depressaria floridella* Mann, 1864	Depressariidae
*Depressaria hirtipalpis* Zeller, 1854	Depressariidae
*Watsonalla uncinula* (Borkhausen, 1790) *	Drepanidae
*Dysauxes parvigutta* (Christoph, 1889)	Erebidae
*Schrankia costaestrigalis* (Stephens, 1834)	Erebidae
*Psoricoptera gibbosella* (Zeller, 1839)	Gelechiidae
*Scrobipalpa bradleyi* Povolný, 1971	Gelechiidae
*Scrobipalpa voltinella* (Chrétien, 1898)	Gelechiidae
*Eupithecia antalica* Mironov, 2001	Geometridae
*Phigalia pilosaria* (Denis & Schiffermüller, 1775)	Geometridae
*Aspilapteryx tringipennella* (Zeller, 1839)	Gracillariidae
*Caloptilia coruscans* (Walsingham, 1907)	Gracillariidae
*Phyllonorycter lapadiella* (Krone, 1909)	Gracillariidae
*Phyllonorycter mespilella* (Hübner, 1805)	Gracillariidae
*Stigmella nivenburgensis* (Preissecker, 1942)	Nepticulidae
*Ctenoplusia limbirena* (Guenée, 1852)	Noctuidae
*Euxoa zernyi* Boursin, 1944	Noctuidae
*Bryophilopsis roederi* (Standfuss, 1892) *	Nolidae
*Garella nilotica* (Rogenhofer, 1882)	Nolidae
*Meganola impura* (Mann, 1862)	Nolidae
*Xanthodes albago* (Fabricius, 1794) *	Nolidae
*Hellinsia carphodactyla* (Hübner, 1813)	Pterophoridae
*Aphomia sabella* (Hampson, 1901) *	Pyralidae
*Epischnia asteris* Staudinger, 1870	Pyralidae
*Euzophera lunulella* (Costa, 1836)	Pyralidae
*Euzopherodes lutisignella* (Mann, 1869)	Pyralidae
*Stemmatophora combustalis* (Fischer von Röslerstamm, 1841)	Pyralidae
*Scythris apicalis* (Zeller, 1847)	Scythrididae
*Stathmopoda auriferella* (Walker, 1864)	Stathmopodidae
*Gaedikeia kokkariensis* Sutter, 1998	Tineidae
*Nemapogon arenbergeri* Gaedike, 1986	Tineidae
*Cnephasia oxyacanthana* (Herrich-Schäffer, 1851)	Tortricidae
*Cochylimorpha decolorella* (Zeller, 1839) *	Tortricidae
*Cochylimorpha diana* (Kennel, 1899)	Tortricidae
*Cochylimorpha langeana* (Kalchberg, 1898)	Tortricidae
*Cydia duplicana* (Zetterstedt, 1839)	Tortricidae
*Endothenia oblongana* (Haworth, 1811)	Tortricidae
*Epiblema graphana* (Treitschke, 1835)	Tortricidae
*Epinotia dalmatana* (Rebel, 1891)	Tortricidae
*Epinotia immundana* (Fischer von Röslerstamm, 1839)	Tortricidae
*Hysterophora maculosana* (Haworth, 1811)	Tortricidae
*Longicornutia epilinana* (Duponchel, 1843)	Tortricidae
*Neocochylis hybridella* (Hübner, 1813)	Tortricidae
*Pammene avetianae* Kuznetsov, 1964	Tortricidae
*Pammene nannodes* Walsingham, 1900	Tortricidae
*Pammene querceti* (Gozmány, 1957)	Tortricidae
*Pseudeulia asinana* (Hübner, 1799)	Tortricidae
*Sparganothis pilleriana* (Denis & Schiffermüller, 1775)	Tortricidae
*Ypsolopha trichonella* (Mann, 1861)	Ypsolophidae

## Data Availability

Detailed data to all 2042 specimens and 1859 COI-5P sequences are available in the dataset “Barcoding Lepidoptera of Cyprus” DS-LEPICYPR on BOLD (https://dx.doi.org/10.5883/DS-LEPICYPR), at https://www.boldsystems.org/ (accessed on 23 November 2025).

## Supplementary Materials

The following supporting information can be downloaded at https://www.mdpi.com/article/10.3390/insects17010004/s1: Table S1: Checklist of Lepidoptera from Cyprus; Table S2: Unidentified BINs; Table S3: New faunistic species records for Cyprus based on DNA barcodes; Table S4: New faunistic species records for Cyprus based solely on morphology; Table S5: Unidentified records based solely on morphology; Table S6: Incorrect or doubtful species records. References [71,72,73,74,75,76,77,78,79,80,81,82,83,84,85,86,87,88,89,90,91,92,93,94,95,96,97,98,99,100,101,102,103,104,105,106,107,108,109,110,111,112,113,114,115,116,117,118,119,120,121,122,123,124,125,126,127,128,129,130,131,132,133,134,135,136,137,138,139,140,141,142,143,144,145,146,147,148,149,150,151,152,153,154,155,156,157,158,159,160,161,162,163,164,165,166,167,168,169,170,171,172,173] are cited in the Supplementary Materials.
